# Fibulin-1C, C1 Esterase Inhibitor and Glucose Regulated Protein 75 Interact with the CREC Proteins, Calumenin and Reticulocalbin

**DOI:** 10.1371/journal.pone.0132283

**Published:** 2015-07-10

**Authors:** Gry Aune Westergaard Hansen, Maja Ludvigsen, Christian Jacobsen, Claudia Cangemi, Lars Melholt Rasmussen, Henrik Vorum, Bent Honoré

**Affiliations:** 1 Department of Biomedicine, Ole Worms Allé 3, Building 1182, Aarhus University, DK-8000 Aarhus C, Denmark; 2 Department of Clinical Biochemistry and Pharmacology, Center for Individualized Medicine in Arterial Diseases (CIMA), Odense University Hospital, Sdr. Boulevard 29, DK-5000 Odense C, Denmark; 3 Department of Ophthalmology, Aalborg University Hospital, Hobrovej 18–22, 9100 Aalborg, Denmark; Russian Academy of Sciences, Institute for Biological Instrumentation, RUSSIAN FEDERATION

## Abstract

Affinity purification, immunoprecipitation, gel electrophoresis and mass spectrometry were used to identify fibulin-1C, C1 esterase inhibitor and glucose regulated protein 75, grp75, as binding partners of the CREC proteins, calumenin and reticulocalbin. Surface plasmon resonance was used to verify the interaction of all three proteins with each of the CREC proteins. Fibulin-1C interacts with calumenin and reticulocalbin with an estimated dissociation constant around 50-60 nM. The interaction, at least for reticulocalbin, was not dependent upon the presence of Ca^2+^. C1 esterase inhibitor interacted with both proteins with an estimated dissociation constant at 1 μM for reticulocalbin and 150 nM for calumenin. The interaction, at least for calumenin, was dependent upon the presence of Ca^2+^ with strong interaction at 3.5 mM while no detectable interaction could be found at 0.1 mM. Grp75 binds with an affinity of approximately 3-7 nM with reticulocalbin as well as with calumenin. These interactions suggest functional participation of the CREC proteins in chaperone activity, cell proliferation and transformation, cellular aging, haemostasis and thrombosis as well as modulation of the complement system in fighting bacterial infection.

## Introduction

The CREC family consists of a set of multiple EF-hand Ca^2+^-binding proteins [1,2]. The family includes at present reticulocalbin [3], ERC-55 and its splice variants [4,5], reticulocalbin-3 [6], Cab45 and its splice variants [2,7,8] as well as calumenin that is now known to exist as 15 splice variants [2,9–13]. The main localization is in the secretory pathway with few exceptions, where Cab45-C and ERC-55-C are present in the cytosol [2,8] and calumenin-15 shuttles between the cytosol and the nucleus [13]. Few members are found to be secreted out of the cell, calumenin [14,15], a Cab45 variant, Cab45-S [2,16,17] and reticulocalbin which may be found on the cell surface [18].

The functional properties are steadily emerging with links to malignant cell transformation [10,19–22] and stress responses [23,24]. A number of molecular functions have been described especially in connection with calumenin in the endoplasmic reticulum (ER) or sarcoplasmic reticulum (SR) [2,25]. Thus, calumenin interacts with the ryanodine receptor-1 possibly regulating its activity [26]. Also, the interaction with and the inhibition of the vitamin K-dependent γ-carboxylation system has been discovered [27] and a polymorphism in the calumenin gene is reported to influence the dose requirements for warfarin anticoagulation [28]. Recently, calumenin was found to interact with the G551D cystic fibrosis transmembrane conductance regulator (CFTR) protein that is causing cystic fibrosis [29]. Calumenin-15 facilitates filopodia formation by increasing GDF-15 transcription [13]. Besides these intracellular functions a few extracellular functions have been reported by the observation that thrombin activated thrombocytes release calumenin which also may be found in atherosclerotic lesions [30]. In vitro experiments have shown that calumenin may modulate the protein expression of fibroblasts [31]. Most recently, calumenin was found to suppress ERK1/2 signalling and cell migration by protecting fibulin-1 from MMP-13-mediated proteolysis [32].

In order to further reveal functional properties of the CREC proteins we searched for novel protein ligands that may interact with two of the family members, calumenin and reticulocalbin. By column immobilization we purified two proteins against each CREC member, fibulin-1C and C1 esterase inhibitor. By immunoprecipitation of cellular proteins and surface plasmon resonance (SRP) analysis the glucose regulated protein 75, grp75, was identified as interacting partner with calumenin and reticulocalbin. The functional implications of these interactions are further discussed.

## Materials and Methods

### Plasmid constructs

We have previously described the expression plasmids consisting of pT7-PL with calumenin [10] and pGEX-4T3 with calumenin [33]. The pGEX expression plasmid containing reticulocalbin was a kind gift of Dr. Ozawa [3] and is also described in [33]. The nucleotides of fibulin-1C (IMAGE clone no. 1174460) encoding amino acids 33–683 of fibulin-1C were cloned into the pGEX-4T3 vector. The subcloned cDNA was PCR amplified using the forward primer: 5’-CCC-GAA-TTC-CGA-TGT-CCT-CCT-GGA-GGC-CTG-CT-3’ and the reverse primer: 5’-CCG-CTC-GAG-TCA-GAG-CTC-TGC-AGA-CAC-CAC-AAA-GAT-G-3’. Thus, the N-terminal amino acids 1–32 that includes the signal sequence are not contained in the expressed protein. The plasmids were transformed into competent *E*. *coli* cells (DH5α, XL1-Blue or BL21-Codon plus). For expression in MRC-5 V2 cells fibulin-1C (IMAGE clone no. 1174460) was PCR amplified using the forward primer: 5’-CCC-TCT-AGA-CCC-GCC-GCC-CAT-GGA-G-3’ and the reverse primer: 5’-CCG-CTC-GAG-TCA-GAG-CTC-TGC-AGA-CA-3’. The cDNA was subcloned into pcDNA3.1/zeo(-). PWO DNA polymerase was used for PCR amplification. The plasmids were checked for errors by DNA sequencing using the service facility at MWG Biotech AG (Ebersberg, Germany).

### Protein expression and purification

Expression and purification of calumenin encoded by the pT7-PL vector was performed as previously described [10]. In short, E. coli cells were re-suspended in lysis buffer (8 M urea, 100 mM NaH_2_PO_4_, 10 mM Tris-HCl, pH 8) and added to a Ni^2+^-NTA-agarose column. The Hexa His-calumenin proteins were eluted in elution buffer (lysis buffer at pH 4.5). The pH was adjusted to 8.0 and dithiotreitol was added to a concentration of 100 mM. GST fusions proteins were expressed in *E*. *coli* after induction with IPTG. The cells were harvested and re-suspended in 1x PBS buffer (2.7 mM KCl, 1.8 mM KH_2_PO_4_, 10.1 mM Na_2_HPO_4_, 140 mM NaCl, pH 7.3). Protese inhibitor cocktail, Complete EDTA free from Roche diagnostic, was added and the cells were lysed by sonication and 20% Triton-x-100 treatment. The supernatant was mixed with glutathione-sepharose beads (Pharmacia). The GST fusion protein were eluted form the beads with glutathione elution buffer (10 mM gluthathione, 50 mM Tris-HCl, pH 8.0). The GST-reticulocalbin fusion protein was cut with thrombin beads from Sigma overnight. The proteins were dialyzed against 1 x PBS (dialysis tubing 8/32 from KemEnTec, diameter 6 mm, cut off 12–14 kDa) in order to remove glutathione. After dialysis glutathione-sepharose beads were added to remove GST from the solution. The supernatant finally contains the recombinant protein.

Eluted proteins from several protein expressions and purifications were pooled, concentrated and furthermore purified by gel filtration on a Superdex 200 column. The protein concentration was measured with a Non-Interfering Protein Assay Kit from Calbiochem or measured spectrophotometrically at 280 nm. Commercially available C1 esterase inhibitor was obtained from Calbiochem. Recombinant GST-grp75 (AAH00478.1) was obtained from Abnova (Taipei, Taiwan).

### Antibodies

The purified recombinant reticulocalbin and calumenin were used for immunization of rabbits and for affinity purification of anti-calumenin and anti-reticulocalbin polyclonal antibodies essentially as previously described [14]. Rabbit polyclonal antibody H-190 was obtained from Santa Cruz Biotechnology, which recognizes an epitope common to all fibulin-1 isoforms (A-D). Monoclonal anti-fibulin-1 antibody was kindly provided by Dr. W. Scott Argraves, Medical University of South Carolina. Monoclonal anti-grp75 antibody was obtained from Biosite.

### Immunoprecipitation

MRC-5 V2 cells were grown in Dulbeccos modified Eagles media (DMEM) with GlutaMAX I containing 10% (v/v) FBS and antibiotic (100 units/ml penicillin and 100 μg/ml streptomycin). Labelling of cells in vivo with L-[^35^S]–Met and L-[^35^S]–Cys were done in labelling medium with 2.5 ml DMEM, 41.5 ml DMEM without methionin, cystein and glutamine, 0.5 ml GlutaMAX I, 5ml dialyzed FBS and 0.5 ml pencilin/streptomycin. Incubation over night in 30 mm tissue cluture test plates from TPP (Trasadingen, Switzerland) with 3 ml labelling medium containing 0.1 mCi/ml of Pro-mix (SJQ 0079) from GE Healthcare. The cells were dissolved in lysis buffer (150 mM NaCl, 5 mM CaCl2, 50 mM Tris-Hcl, pH 7.4, 0.5% Triton x-100, 0.5% Na-deoxycholate). Antiserum, 6.25 mg/ml BSA, 1 x NCTTAM buffer (150 mM NaCl, 5 mM CaCl_2_, 50 mM Tris-HCl, pH 7.4, 1% Triton x-100, 1 mg/ml BSA, 20 mM methionin, 0.02% NaN_3_) were added to the cell lysate and incubated for one hour at room temperature. Immunocomplexes were pelleted by incubation with GammaBind G Sepharose beads from GE Healthcare for 30 minutes and centrifuged. The pellets were washed twice with 1 x NCTTAM buffer and twice with ice-cold 2 times distilled H_2_O and the immunocomplexes were eluted with 2 x Laemmli buffer (0.125 M Tris-HCl, pH 6.8, 20% glycerol, 4% SDS, 0.005% bromophenol blue) for 10 minutes at 95°C followed by centrifugation. The immunocomplexes were precipitated with ethanol and used for 2D-PAGE analyses.

### Affinity purification of reticulocalbin and calumenin interacting proteins from human placenta

Recombinant reticulocalbin and calumenin from the protein expressions were dialyzed in coupling buffer (0.1 M NaHCO_3_, 0.5 M NaCl, pH 8.3) and then concentrated in centriplus 3 and centricon YM-3 tubes from Amicon. CNBr activated sepharose 4B was swelled and washed with 200 ml 1 mM HCl and then mixed with coupling buffer (reference column) and concentrated reticulocalbin and calumenin, respectively. After the coupling excess ligand was washed away from the gel with coupling buffer and blocking buffer (0.1 M Tris-HCl, pH 8.0) was added to block any remaining active groups. Afterwards the gel matrix was washed in buffers with three cycles of alternating pH to completely remove excess of uncoupled ligand. Each cycle consisted of a wash with washing buffer 1 (0.1 M Na-acetat, 0.5 M NaCl, pH 4.0) followed by a wash with washing buffer 2 (0.1 M Tris, 0.5 M NaCl, pH 8.0). The columns were packed with the gel matrix equilibrated in MB-buffer (2 mM CaCl_2_, 1 mM MgCl_2_, 10 mM HEPES, 140 mM NaCl, pH 7.4). Equal volumes of dialyzed placenta extract prepared as previously described [33] were added to the columns with immobilized recombinant reticulocalbin and calumenin and the reference columns without reticulocalbin and calumenin, respectively. The columns were then washed in MB-buffer before elution with MB-buffer containing 200 mM, 400 mM, 600mM, 800 mM, 1000 mM NaCl and finally 1000 mM NaCl with EDTA (10 or 20 mM), 10 mM HEPES, pH 7.4. The eluates were up-concentrated and analyzed for potential protein ligands by gel electrophoresis and subsequently identified by mass spectrometry.

### Gel electrophoresis

One-dimensional (1D) and two-dimensional (2D) polyacrylamide gel electrophoresis (PAGE) as well as silver staining were essentially performed as previously described [34].

### Cell culture and transfection assay

MRC-5 V2 cells were grown as monolayer cultures in DMEM supplemented with 10% fetal bovine serum and antibiotics (100 U/ml pencilin and 50 γg/ml streptomycin). To establish transfected cell lines overexpressing fibulin-1C, plasmid encoding fibulin-1C in pcDNA3.1/zeo(-) was transfected into human MRC-5 V2 fibroblast cells using FuGENE 6 transfection reagent (Roche Diagnostics).

### Mass spectrometry

Gels containing protein spots were re-swelled in water and the protein spots of interest were cut out and treated as previously given [33].

### Immunofluorescence microscopy

MRC-5 V2 cells were grown on coverslips to 60–70% confluence. Then the cells were fixed for 10 min with 3.7% formalin and subsequently washed with 1 x PBS. The cells were permeabilized with 0.1% Triton X-100 and 3.7% formalin and washed again. The formalin fixed MRC-5 V2 cells were double stained with rabbit anti-reticulocalbin antibody, rabbit anti-calumenin antibody, mouse anti-grp75 or mouse anti-fibulin-1 mAb 3A11 for one hour at 37°C in a moist chamber. The cells were washed repeatedly in 1 x PBS and incubated with donkey anti rabbit IgG (Alexa Flour 594 conjugated) or donkey anti mouse IgG (alexa flour 488 conjugated) secondary antibody diluted 1:5000 for one hour at room temperature. For nucleus staining a DAPI solution diluted 1:50000 was added and incubated for 10 min. Upon washing the cells were mounted with Fluorescence mounting medium from DAKO.

### Immunohistochemistry

Normal appearing, non-atherosclerotic internal mammary artery biopsies, collected at coronary bypass surgery as described before [35], and inner lining of atherosclerotic carotid artery, collected at endarterectomy surgeries, were employed for immunohistological analyses. Upon collection, the specimens were fixed in PBS (10 mmol/L phosphate, 150 mmol/L NaCl, pH 7.2), buffered 4% formaldehyde and subsequently embedded in paraffin. Sections of arterial samples (4 μm) were incubated overnight at 4°C with rabbit polyclonal antibody H-190 (Santa Cruz Biotechnology, Inc.; dil.:1/100), which recognizes an epitope common to all fibulin-1 isoforms (A-D). Envision system HRP-conjugated secondary antibodies were used (K4002, Dako) for detection. Diaminobenzidine (DAB+, K3468; Dako) was applied for 50 seconds and stopped in H_2_O. Control sections were incubated with secondary antibody only. Caluminin and reticulocalbin staining was done with rabbit polyclonal antibodies affinity purified and tested as previously described [14] using UltraView Universal Alkaline Phosphatase Red Detection Kit from Ventana Medical Systems.

### Surface plasmon resonance (SPR)

Interaction analyses were performed using Biacore 2000/3000 instrument using CM5 sensor chips (GE Healthcare). Amine-coupling reagents (*N*-ethyl-*N*’-(3-dimethylaminopropyl) carbodiimide and *N*-hydroxysuccinimide were used for activation of the sensor chip, creating a reactive ester on the surface. After reticulocalbin and calumenin immobilization, the sensor surface was deactivated with 1 M ethanolamine, pH 8.5, to block the remaining unreacted groups. Fibulin-1C and C1 esterase inhibitor were dissolved with running buffer (10 mM HEPES, 150 mM NaCl, 5 mM CaCl_2_, 1mM EGTA and 0.005% Tween 20, pH 7.4), and binding experiments were performed at 25°C in running buffer. A control flow cell with an activated but uncoupled chip was used as a reference to subtract nonspecific binding. Regeneration of the sensor chip after each analysis cycle was performed with 10 mM glycine, 20 mM EDTA, 500 mM NaCl and 0.005% Tween 20, pH 4.0. Due to high affinity interaction this buffer was not sufficient to remove grp75 from the chips. Instead a buffer containing 0.05% SDS was needed to remove grp75. Sensorgrams show SPR signal against time and is expressed in relative response units as the difference in response between the flow cell with immobilized calumenin or reticulocalbin and a control flow cell. Data were analyzed by global fitting to a 1:1 Langmuir binding model of both the association and dissociation phases for one or several concentrations, using BIAevaluation 4.1 software (GE Healthcare). Apparent equilibrium dissociation constant (*K*_D_) was calculated from the ratio of the dissociation and association rate constants (*k*_off_/*k*_on_).

### Tissue

The local ethical committee of the County of Aarhus and the regional committee for Biomedical Research Ethics of Southern Denmark (approval number S-20140202) approved the use of human tissue. All patients signed informed consent.

## Results

### Affinity purification of calumenin and reticulocalbin interacting proteins from placenta

**Fig 1:**
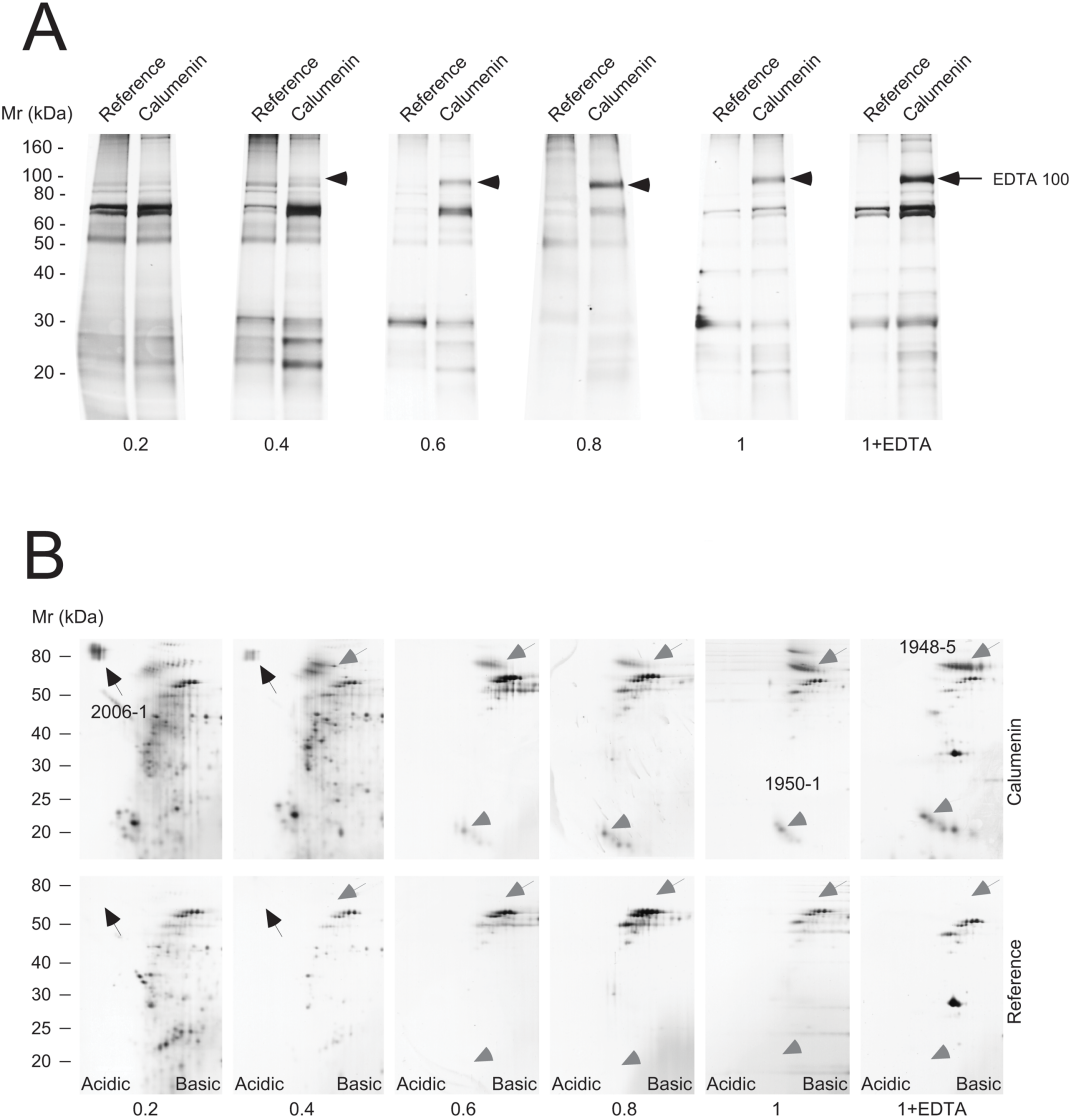
Affinity purification of calumenin binding proteins from extract of placenta. Equal volumes of homogenized human placenta were applied to a column with immobilised calumenin and to a column without calumenin. Both columns were eluted with stepwise increasing concentrations of NaCl from 0.2 M to 1 M as indicated and finally with 1M NaCl containing 20 mM EDTA. Equal volumes of concentrated samples were applied to 1D-PAGE (A) and 2D-PAGE (B) gel electrophoresis with subsequent silver staining. The acidic side and the basic side of the first dimensions of the 2D gels are indicated. Several proteins were eluted from the calumenin column as well as the reference column. The band marked EDTA 100 and the spots marked 2006–1, 1948–5 and 1950–1 were excised from the rehydrated gels and subjected to mass spectrometry identification as given in Table 1.

**Fig 2:**
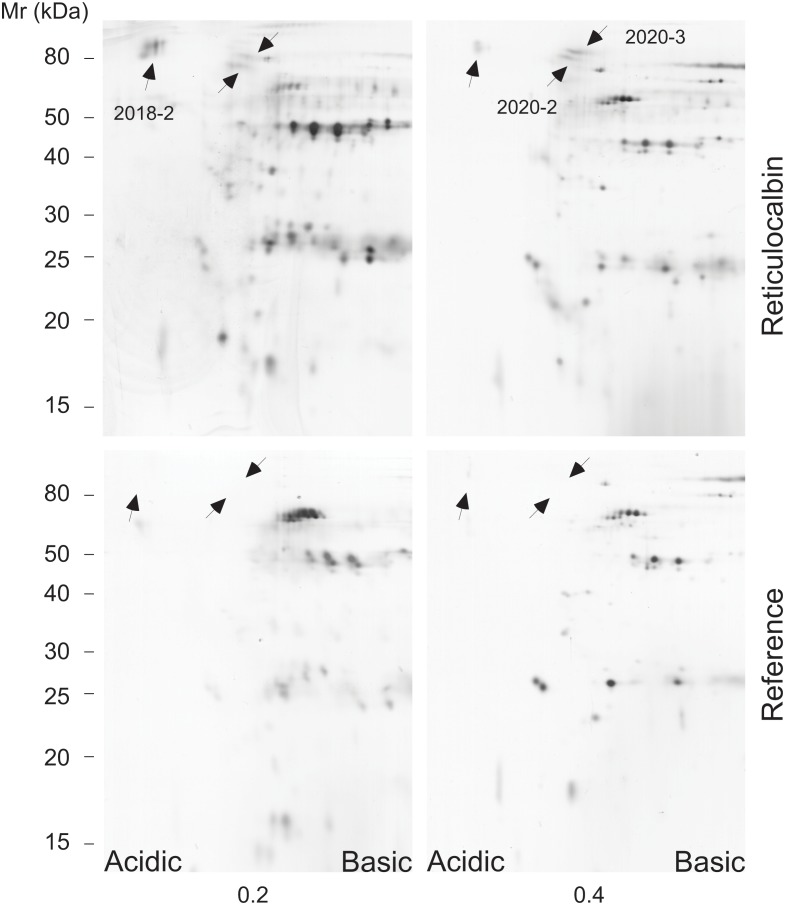
Affinity purification of reticulocalbin binding proteins from extract of placenta. Equal volumes of homogenized placenta were applied to a column with immobilised reticulocalbin and to a column without reticulocalbin. Elution was performed similarly as described to Fig 1. Equal volumes of concentrated samples were applied to 2D gel electrophoresis with subsequent silver staining. The acidic side and the basic side of the first dimensions of the 2D gels are indicated. Several spots were eluted from the reticulocalbin column as well as the reference column. The spots marked 2018–2, 2020–2 and 2020–3 were excised from the rehydrated gels and subjected to mass spectrometry identification as given in Table 1.

Several liters of placenta extract were equally applied to a column with immobilized calumenin and a parallel column without coupled calumenin. After washing in several column volumes of MB-buffer with 140 mM NaCl the proteins from each of the columns were eluted with a step-wise gradient using increasing concentrations of NaCl from 0.2 M to 1 M and finally 1 M NaCl with the inclusion of EDTA. Eluates were concentrated and analyzed by 1D-PAGE (Fig 1A) or 2D-PAGE (Fig 1B). Apparently, the columns bind a number of proteins non-specifically. However, one band around 80–100 kDa was seen faintly in the 0.4 M fraction and with increasing intensity using steadily higher salt concentrations and it also appeared in the elution containing EDTA. The band marked (EDTA 100) was excised from the gel and subjected to *in gel* tryptic digestion. MALDI-TOF analysis revealed that the band contained fibulin-1 (Table 1). The band also contained traces of thrombospondin-1, which we previously have identified as a calumenin interacting protein [33]. We further analyzed the fractions by 2D-PAGE as seen in Fig 1B. Although a number of unspecific proteins also are apparent with this analysis clearly a similar elution pattern could be seen. A set of co-migrating protein spots were found around 80 kDa in the elution from the calumenin column which was clearly absent from the reference column (gray arrows, Fig 1B). In addition to the 80-kDa co-migrating proteins a string of spots around 20 kDa was eluted from the calumenin column using increasing concentrations of NaCl and also appeared in the elution containing EDTA (gray arrowheads, Fig 1B). As indicated in Fig 1B representatives of these proteins were excised from the gels for identification by mass spectrometry (1948–5 and 1950–1). The representatives revealed that the 80-kDa protein was fibulin-1 and the 20 kDa protein apparently an N-terminal fragment of fibulin-1 (Table 1). From the 2D-PAGE analysis it was further apparent that one diffuse spot revealed a quite different elution profile. The spot is located around 80 kDa in an acidic position (pI ≈ 3 to 4). The spot was found in the elutions containing 0.2 and 0.4 M NaCl and was not apparent at elutions containing higher salt concentration. This spot was excised from the gels containing the elution with 0.2 M NaCl (Spot No. 2006–1). The MALDI TOF analysis revealed that the protein was C1 esterase inhibitor (Table 1).

**Table 1:** Mass spectrometric identification of CREC interacting proteins.

Protein spot	Measured mass	Computedmass	Peptides identified by MALDI	Position of peptide in protein	Peptide coverage	Protein identification
EDTA 100	1989.888	1989.916	IIEVEEEQEDPYLNDR	164–179	16%	Fibulin 1, CAQ09097.1^b^ (1–683 amino acids)
	1174.566	1174.536	GYHLNEEGTR	344–353		
	1177.560	1177.540	TGYYFDGISR	386–395		
	2336.247	2336.231	ITYYHLSFPTNIQAPAVVFR	586–605		
	2856.376	2856.304	MGPSSAVPGDSMQLAITGGNEEGFFTTR	606–633		
	1997.106	1997.141	KVSPHSGVVALTKPVPEPR	634–652		
	800.477	800.500	DLLLTVK	653–659		
1948–5	1989.910	1989.916	IIEVEEEQEDPYLNDR	164–179	21	Fibulin 1, CAQ09097.1 (1–683 amino acids)
	1438.648	1438.687	LGESCINTVGSFR	229–241		
	815.354	815.370	NVPNCGR	337–343		
	1174.551	1174.536	GYHLNEEGTR	344–353		
	1022.468	1022.460	VCNSPGSFR	373–381		
	1177.550	1177.540	TGYYFDGISR	386–395		
	1309.516	1309.521	MCVDVNECQR	396–405		
	1457.589	1457.606	CLAFECPENYR	551–561		
	1369.599	1369.597	CERLPCHENR	568–577		
	2336.269	2336.231	ITYYHLSFPTNIQAPAVVFR	586–605		
	2856.411	2856.304	MGPSSAVPGDSMQLAITGGNEEGFFTTR	606–633		
	2337.240^a^		ITYYHLSFPTNIQAPAVVFR			
1950–1	1269.561	1269.554	DCSLPYATESK	49–59	5	Fibulin 1, CAQ09097.1 (1–683 amino acids)
	1234.521	1234.482	CCHCCLGR	109–117		
	1790.851	1790.816	SQETGDLDVGGLQETDK	147–163		
2006–1	1263.670	1263.670	TNLESILSYPK	191–201	21	C1 inhibitor, BAF85743 (1–500 amino acids)
	1217.584	1217.586	DFTCVHQALK	202–211		
	1825.916	1825.968	GVTSVSQIFHSPDLAIR	217–233		
	909.458	909.455	TLYSSSPR	242–249		
	1115.581	1115.582	LLDSLPSDTR	277–286		
	1191.604	1191.585	TRMEPFHFK	308–316		
	1430.744	1430.755	YPVAHFIDQTLK	330–341		
	1885.859	1885.935	HRLEDMEQALSPSVFK	365–380		
	1184.698	1184.691	FQPTLLTLPR	391–400		
	1185.700^a^		FQPTLLTLPR			
2018–2	1263.644	1263.670	TNLESILSYPK	191–201	23	C1 inhibitor, BAF85743 (1–500 amino acids)
	1217.593	1217.586	DFTCVHQALK	202–211		
	1825.884	1825.968	GVTSVSQIFHSPDLAIR	217–233		
	909.452	909.455	TLYSSSPR	242–249		
	1115.589	1115.582	LLDSLPSDTR	277–286		
	1191.590	1191.585	TRMEPFHFK	308–316		
	1558.807	1558.850	KYPVAHFIDQTLK	329–341		
	1885.866	1885.935	HRLEDMEQALSPSVFK	365–380		
	1210.605	1210.593	AIMEKLEMSK	381–390		
	1184.694	1184.691	FQPTLLTLPR	391–400		
	1185.670^a^		FQPTLLTLPR			
2020–2	1234.494	1234.482	CCHCCLLGR	109–117	26	Fibulin 1, CAQ09097.1 (1–683 amino acids)
	1989.911	1989.916	IIEVEEEQEDPYLNDR	164–179		
	1438.656	1438.687	LGESCINTVGSFR	229–241		
	815.365	815.370	NVPNCGR	337–343		
	1174.552	1174.536	GYHLNEEGTR	344–353		
	2152.887	2152.907	CVDVDECAPPAEPCGKGHR	354–372		
	1022.462	1022.460	CVNSPGSFR	373–381		
	1177.549	1177.540	TGYYFDGISR	386–395		
	3386.602	3386.447	GYQLSDVDGVTCEDIDECALPTGGHICSYR	468–497		
	1457.600	1457.606	CLAFECPENYR	551–561		
	1369.576	1369.597	CERLPCHENR	568–577		
	924.423	924.423	LPCHENR	571–581		
	2336.115	2336.231	ITYYHLSFPTHIQAPAVVFR	586–605		
2020–3	1989.875	1989.916	IIEVEEEQEDPYLNDR	164–179	17	Fibulin 1, CAQ09097.1 (1–683 amino acids)
	1438.671	1438.687	LGESCINTVGSFR	229–241		
	1174.558	1174.536	GYHLNEEGTR	344–353		
	1022.477	1022.460	CVNSPGSFR	373–381		
	1177.537	1177.540	TGYYFDGISR	386–395		
	1457.696	1457.606	CLAFECPENYR	551–561		
	1369.581	1369.597	CERLPCHENR	568–577		
	2336.293	2336.231	ITYYHLSFPTNIQAPAVVFR	586–605		
	1997.050	1997.141	KVSPHSGVVALTKPVPEPR	634–652		
Protein 2	1682.83	1682.75	HQDSWNGLSHEAFR	26–39	41	Grp75, AAA67526
	2092.90	2093.01	GAVVGIDLGTTNSCVAVMEGK	53–73		
	1449.71	1449.71	TTPSVVAFTADGER	86–99		
	1568.81	1568.83	LYSPSQIGAFVLMK	160–173		
	1607.80	1607.76	MKETAENYLGHTAK	174–187		
	1693.84	1693.84	NAVITVPAYFNDSQR	188–202		
	1669.89	1669.91	QATKDAGQISGLNVLR	203–218		
	1241.68	1241.67	DAGQISGLNVLR	207–218		
	1644.87	1644.87	VINEPTAAALAYGLDK	219–234		
	2054.94	2054.95	STNGDTFLGGEDFDQALLR	266–284		
	1705.82	1705.83	ETGVDLTKDNMALQR	293–307		
	1360.74	1360.74	AQFEGIVTDLIR	349–360		
	1461.75	1461.75	SDIGEVILVGGMTR	378–391		
	1289.68	1289.67	VQQTVQDLFGR	395–405		
	1807.89	1807.90	SQVFSTAADGQTQVEIK	469–485		
	1591.95	1591.94	LLGQFTLIGIPPAPR	499–513		
	1420.69	1420.65	DDIENMVKNAEK	556–567		
	2157.02	2157.04	ERVEAVNMAEGIIHDTETK	577–595		
	1871.89	1871.89	VEAVNMAEGIIHDTETK	579–595		
	2232.07	2232.10	DSETGENIRQAASSLQQASLK	626–646		
Protein 3	1682.82	1682.75	HQDSWNGLSHEAFR	26–39	40	Heat shock 70 kDa protein 9 (mortalin), AAH00478
	1156.58	1156.62	AKVLENAEGAR	75–85		
	1708.78	1708.86	IRASNGDAWVEAHGK	144–159		
	1693.83	1693.84	NAVITVPAYFNDSQR	188–202		
	1669.88	1669.91	QATKDAGQISGLNVLR	203–218		
	1241.66	1241.67	DAGQISGLNVLR	207–218		
	1644.85	1644.87	VINEPTAAALAYGLDK	219–234		
	2054.93	2054.95	STNGDTFLGGEDFDQALLR	266–284		
	1705.81	1705.83	ETGVDLTKDNMALQR	293–307		
	1360.71	1360.74	AQFEGIVTDLIR	349–360		
	2437.19	2437.18	AMQDAEVSKSDIGEVILVGGMTR	369–391		
	1461.73	1461.75	SDIGEVILVGGMTR	378–391		
	1289.65	1289.67	VQQTVQDLFGR	395–405		
	1807.88	1807.90	SQVFSTAADGQTQVEIK	469–485		
	1591.93	1591.94	LLGQFTLIGIPPAPR	499–513		
	2433.22	2433.21	EQQIVIQSSGGLSKDDIENMVK	542–563		
	1420.69	1420.65	DDIENMVKNAEK	556–567		
	1871.89	1871.89	VEAVNMAEGIIHDTETK	579–595		
	2232.08	2232.10	DSETGENIRQAASSLQQASLK	626–646		
	1044.56	1044.53	LFEMAYKK	647–654		

^a^Mass of parent ion (single charged) used for MALDI-PSD peptide sequencing.

^b^The band contains traces of thrombospondin-1.

A similar analysis was performed of placenta proteins eluted from a reticulocalbin column in parallel with a reference column. Only the 0.2 M NaCl and 0.4 M NaCl elutions are shown (Fig 2). Again several proteins bind unspecifically to the columns. However, although the intensities of the spots are low, one acidic diffuse spot located around 80 kDa (pI ≈ 3 to 4) is especially seen in the elution fraction with 0.2 M NaCl (spot 2018–2) but also seen to some extend in the fraction with 0.4 M NaCl from the reticulocalbin column. The spot was not detected in the corresponding elution fractions from the reference column. Excision of the spot (No. 2018–2) with subsequent MALDI-TOF analysis identified the protein as C1 esterase inhibitor (Table 1). In the 0.4 M NaCl elution fraction several 80 kDa partly co-migrating spots was seen from the reticulocalbin column that were absent from the reference column (spots No. 2020–2 and 2020–3). They were also seen to some extend in the elution fraction using 0.2 M NaCl from the reticulocalbin column but not from the reference column. Both spots were excised for MALDI-TOF analysis and were identified as fibulin-1 (Table 1).

Thus, the affinity purification of placenta proteins using immobilized calumenin as well as immobilized reticulocalbin identified fibulin-1 as an interacting protein with both CREC proteins. Fibulin 1 exists in four variants A, B, C and D and the identified fibulin indicated that the purified protein was of the C type. In addition C1 esterase inhibitor was found to interact with both CREC proteins. In order to minimize artefact protein identifications we focused on spots that were highly enriched in the analytical eluates showing a “consistent” elution pattern by being present in several fractions. SPR was subsequently used to verify these interactions.

### Surface plasmon resonance verification of fibulin-1C interaction with reticulocalbin and calumenin

**Fig 3:**
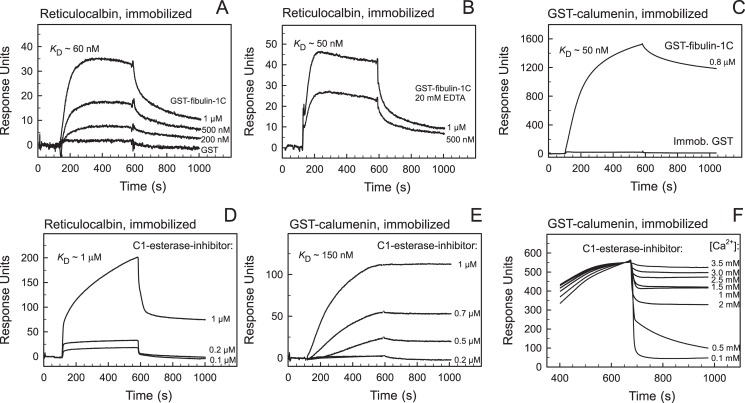
Surface plasmon resonance analysis of fibulin-1C and C1 esterase inhibitor with calumenin and reticulocalbin. (A) Reticulocalbin was immobilised to the chip and various concentrations of GST-fibulin-1C was applied at indicated concentrations in buffer (10 mM Hepes, 150 mM NaCl, 5 mM CaCl_2_, 1 mM EGTA and 0.005% Tween 20, pH 7.4). The dissociation constant is estimated to be approx. 60 nM. GST was also applied to the chip and showed no detectable binding. (B) Various concentrations of GST-fibulin-1C were applied at indicated concentrations in buffer with EDTA (10 mM Hepes, 150 mM NaCl, 20 mM EDTA and 0.005% Tween 20 pH 7.4). The dissociation constant is estimated to be approx. 50 nM. (C) GST-fibulin-1C, 0.8 μM, was applied to a chip with immobilised GST-calumenin and to a chip with immobilised GST. The affinity of GST-fibulin-1C was estimated to approx. 50 nM. The buffer was similar to that given in A. (D) C1 esterase inhibitor was applied in varying concentrations to a chip with immobilized reticulocalbin. The dissociation constant was estimated to be approx. 1 μM. The buffer was similar to that given in A. (E) GST-calumenin was immobilized to a chip and C1 esterase inhibitor was applied in varying concentrations as indicated. The dissociation constant was estimated to approx. 150 nM. The buffer was similar to that given in A. (F) C1 esterase inhibitor was applied to the chip in buffer containing 4 mM free Ca^2+^ (10 mM Hepes, 150 mM NaCl, 5 mM CaCl_2_, 1 mM EGTA and 0.005% Tween 20, pH 7.4). In the dissociation phase the Ca^2+^ concentration was varied from 3.5 mM to 0.1 mM as indicated. No detectable binding was observed at 0.1 mM Ca^2+^. Regeneration of the sensor chips after each analysis cycle was performed with 10 mM glycine, 20 mM EDTA, 500 mM NaCl and 0.005% Tween 20, pH 4.0.

SPR was used to verify the interactions between the identified proteins and immobilized reticulocalbin and calumenin as shown in Fig 3. Various concentrations of recombinant GST-fibulin-1C were applied to the chip with immobilized reticulocalbin in presence of Ca^2+^ (Fig 3A). A large increase in response units is seen with the addition of GST-fibulin-1C in the association phase. The dissociation phase starts around 600 seconds by the addition of buffer. The dissociation constant is estimated to be around 60 nM (*k*_diss_/*k*_ass_). No detectable binding was seen when GST was applied to immobilized reticulocalbin. Inclusion of 20 mM EDTA in the buffer gave an interaction with a similar affinity indicating that the binding of fibulin-1C to reticulocalbin is not dependent upon the presence of Ca^2+^ (Fig 3B).

Fibulin-1C was further added to a GST-calumenin chip (Fig 3C). The dissociation constant was estimated to be around 50 nM and no interaction was seen when GST-fibulin-1C was applied to immobilized GST (Fig 3C). Thus, the interaction between fibulin-1C and reticulocalbin and calumenin was thereby verified. For reticulocalbin the binding was not dependent upon the presence of Ca^2+^.

### Surface plasmon resonance verification of C1 esterase inhibitor interaction with reticulocalbin and calumenin

C1 esterase inhibitor was applied to the reticulocalbin chip in various concentrations between 0.1 and 1 μM as shown in Fig 3D. The affinity was estimated to be around 1 μM with respect to reticulocalbin. By applying C1 esterase inhibitor to the GST-calumenin chip in various concentrations between 0.2 and 1 μM the affinity was estimated to be around 150 nM (Fig 3E). The interaction of C1 esterase inhibitor with GST-calumenin was further analyzed with the presence of 4 mM Ca^2+^ in the association phase and by varying the Ca^2+^ concentration in the dissociation phase stepwise between 3.5 mM and 0.1 mM. At a free Ca^2+^ concentration of 3.5 mM the dissociation rate was very low corresponding to the affinity estimated to approx. 150 nM. By decreasing the Ca^2+^ concentration in the dissociation phase we find a steadily increasing dissociation rate that approaches to an undetectable interaction at a Ca^2+^ concentration around 0.1 mM. Thus, the strong interaction between C1 esterase inhibitor and calumenin is dependent upon the presence of Ca^2+^ in the mM concentration range (0.5 mM to 3.5 mM) with no detectable interaction at 0.1 mM.

### Immunoprecipitation with anti-calumenin and anti-reticulocalbin antiserum

**Fig 4:**
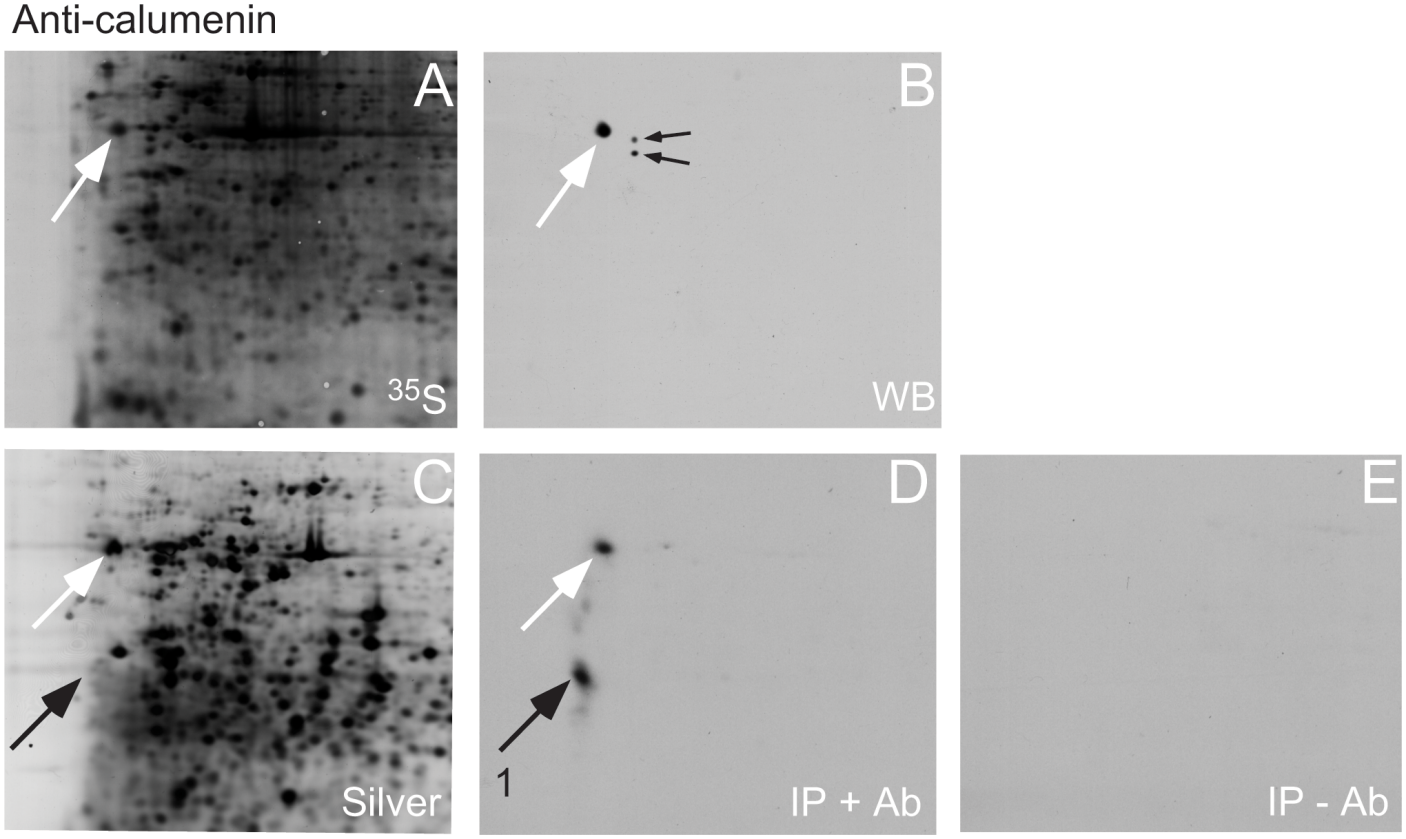
Immunoprecipitation of MRC-5 V2 proteins with anti-calumenin antiserum. (A) ^35^S-labelled MRC-5 V2 proteins were separated by 2D-PAGE, blotted onto a membrane and subjected to autoradiography. The autoradiogram is shown. (B) The membrane was used for Western blotting by incubating with anti-calumenin antiserum. The antibody showed a strong reaction against calumenin and a very slight reaction against two reticulocalbin spots (small black arrows). (C) ^35^S-labelled MRC-5 V2 proteins were immunoprecipitated with anti-calumenin antiserum mixed with unlabelled proteins and separated by 2D-PAGE with subsequent silver staining. (D) Autoradiogram of ^35^S-labelled immunoprecipitated proteins. Calumenin was immunoprecipitated (white arrow) as well as protein 1. (E) No proteins were immunoprecipitated without the presence of anti-calumenin antiserum.

In order to seek for intracellular calumenin and reticulocalbin binding proteins we used immunoprecipitation of MRC-5 V2 fibroblast proteins with polyclonal antisera against calumenin and reticulocabin. Fig 4 shows a 2D-PAGE immunoblot of anti-calumenin antiserum using MRC-5 V2 fibroblast proteins. The antiserum is very specific with a strong reaction against calumenin (white arrow, Fig 4A and 4B). Only a slight reaction is found against two reticulocalbin spots (small black arrows, Fig 4B). Immunoprecipitation was performed using L-[^35^S]-Met and L-[^35^S]-Cys labelled MRC-5 V2 proteins. The immunoprecipitated and labelled proteins were mixed with unlabeled MRC-5 V2 proteins and separated by 2D-PAGE before autoradiography. Fig 4C shows the separated proteins and Fig 4D the autoradiogram. The anti-calumenin antiserum precipitates calumenin (white arrow, Fig 4C and 4D) together with an acidic protein, No. 1 (black arrow, Fig 4C and 4D) while no proteins are precipitated without the presence of anti-calumenin antiserum (Fig 4E). The precipitated protein however did not focus very well in the acidic region of the 2D-PAGE analysis and was present in insufficient amounts for mass spectrometric determination.

**Fig 5:**
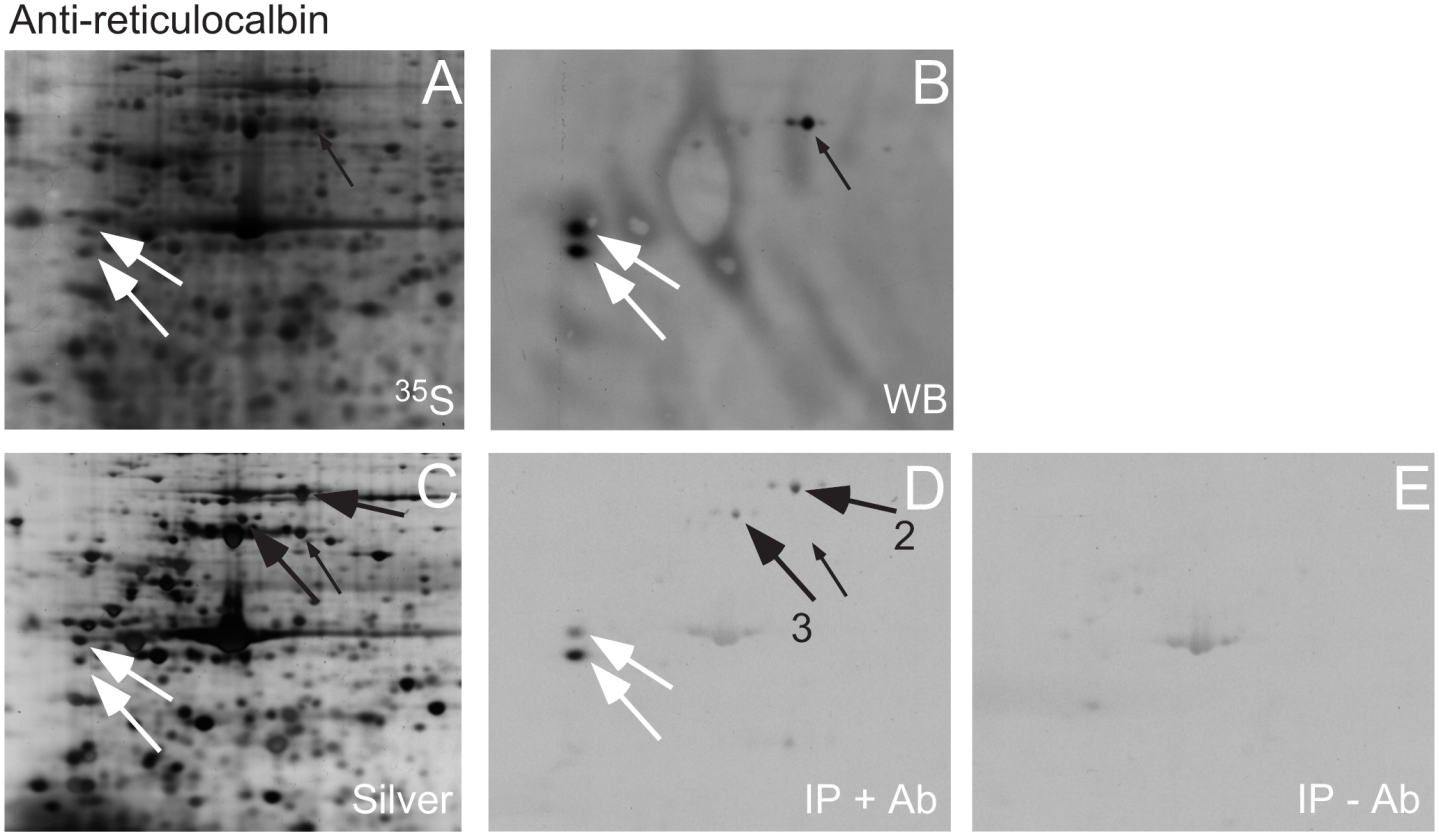
Immunoprecipitation of MRC-5 V2 proteins with anti-reticulocalbin antiserum. (A) ^35^S-labelled MRC-5 V2 proteins were separated by 2D-PAGE, blotted onto a membrane and subjected to autoradiography. The autoradiogram is shown. (B) The membrane was used for Western blotting by incubating with anti-reticulocalbin antiserum. The antibody showed a strong reaction against two reticulocalbin spots (white arrows) and a slight cross-reaction against an unknown protein (small black arrow). (C) ^35^S-labelled MRC-5 V2 proteins were immunoprecipitated with anti-reticulocalbin antiserum mixed with unlabelled proteins and separated by 2D-PAGE with subsequent silver staining. (D) Autoradiogram of ^35^S-labelled, immunoprecipitated proteins. Two reticulocalbin spots were immunoprecipitated (white arrows) as well as protein 2 and 3. Proteins 2 and 3 were identified as grp75 by mass spectrometry (see text and Table 1). The cross-reacting protein was not immunoprecipitated. (E) No proteins were immunoprecipitated without the presence of anti-reticulocalbin antiserum.

A similar immunoprecipitation was performed with anti-reticulocalbin antiserum (Fig 5). Western blot analysis revealed that the antiserum reacted strongly against two reticulocalbin spots (white arrows, Fig 5A and 5B) with a cross-reaction (small black arrow, Fig 5B). Immunoprecipitation was performed using L-[^35^S]-Met and L-[^35^S]-Cys labelled MRC-5 V2 proteins. The immunoprecipitated and labelled proteins were mixed with unlabeled MRC-5 V2 proteins and separated by 2D-PAGE before autoradiography. Fig 5C shows the separated proteins and Fig 5D the corresponding autoradiogram. The anti-reticulocalbin antiserum precipitates the two reticulocalbin spots together with two spots, Nos. 2 and 3 (black arrows, Fig 5D). The anti-reticulocalbin antiserum did not precipitate the cross-reacting spot indicating that the interaction between reticulocalbin and the precipitated proteins may be real. No proteins are precipitated without the presence of anti-reticulocalbin antiserum (Fig 5E). The two spots, No. 2 and 3, were excised from the silver stained 2D-PAGE gel and subjected to in-gel digestion and mass spectrometry analysis. This revealed that spot 2 was identified as grp75 (Accession GI:292059) and spot 3 as heat shock 70 kDa protein 9 (mortalin) (Accession GI:18645123). Except for 3 amino acid changes of 679 amino acids the proteins are listed as identical in the SwissProt database as 75 kDa glucose regulated protein (GRP75_HUMAN).

### Surface plasmon resonance verification of grp75 interaction with calumenin and reticulocalbin

**Fig 6:**
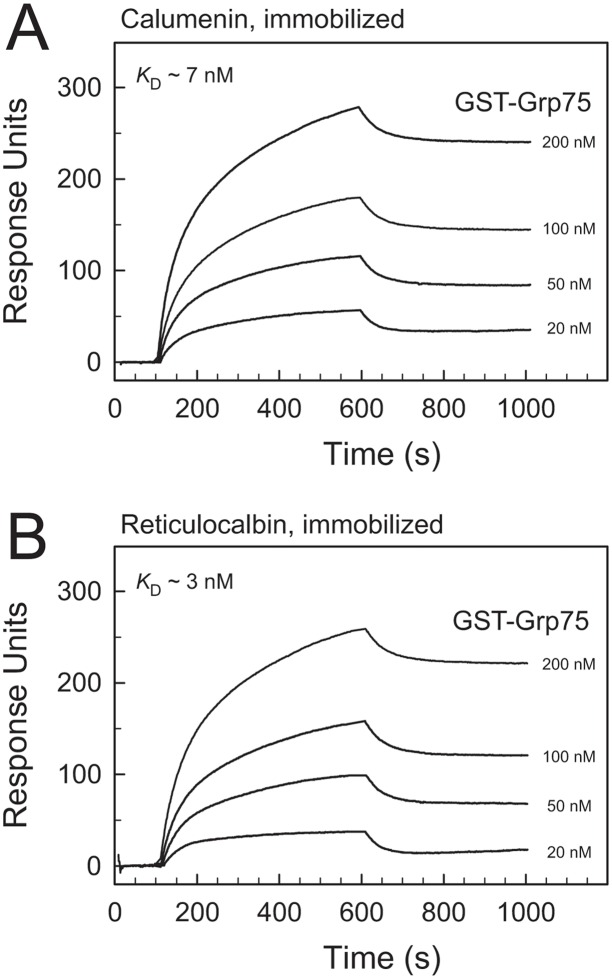
Surface plasmon resonance analysis of grp75 binding to calumenin and reticulocalbin. (A) Calumenin was immobilised to the chip and various concentrations of GST-grp75 (AAH00478.1) were applied at indicated concentrations in buffer with EGTA (10 mM Hepes, 150 mM NaCl, 5 mM CaCl_2_, 1 mM EGTA and 0.005% Tween 20 pH 7.4). The dissociation constant was estimated to be approx. 7 nM. (B) Various concentrations of GST-grp75 (AAH00478.1) were applied at indicated concentrations in buffer with EGTA (10 mM Hepes, 150 mM NaCl, 5 mM CaCl_2_, 1 mM EGTA and 0.005% Tween 20, pH 7.4). The dissociation constant was estimated to be approx. 3 nM.

We used SPR to verify the interaction of GST-grp75 with both CREC proteins immobilized to the chip as shown in Fig 6. Various concentrations of GST-grp75 were applied to the chips with immobilized calumenin and immobilized reticulocalbin. The analysis showed that GST-grp75 interacts strongly with both CREC proteins with affinities in the low nanomolar range.

### Confocal microscopy

**Fig 7:**
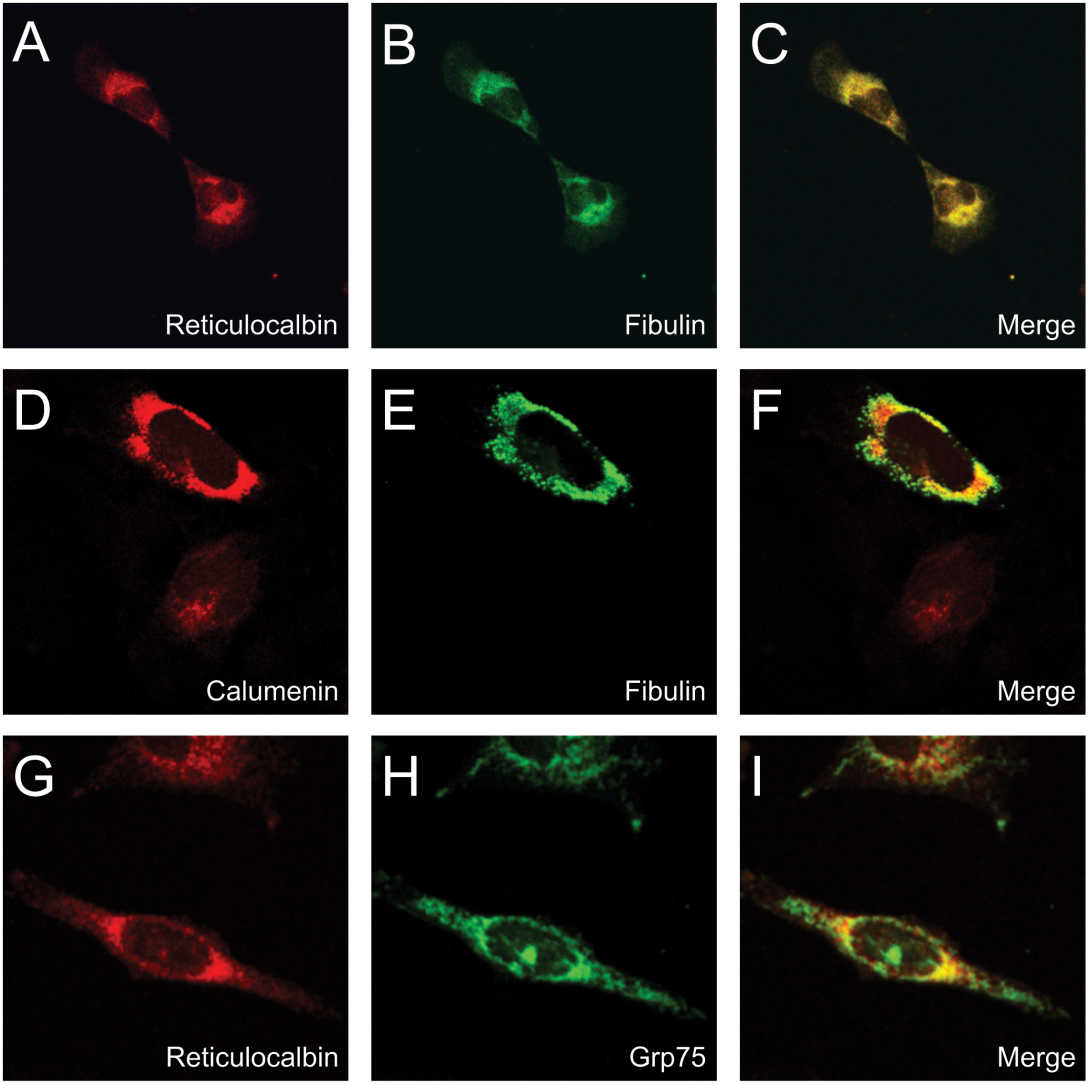
Confocal microscopy of CREC proteins with fibulin and grp75. (A) MRC-5 V2 cells were transfected with fibulin-1C and stained for reticulocalbin (red fluorescence) and (B) fibulin-1C (green fluorescence). (C) The merged picture shows colocalization of reticulocalbin and fibulin-1C (yellow fluorescence). (D) MRC-5 V2 cells were transfected with fibulin-1C and stained for calumenin (red fluorescence) and (E) fibulin-1C (green fluorescence). (F) The merged picture shows colocalization of calumenin and fibulin-1C (yellow fluorescence). (G) MRC-5 V2 cells stained for reticulocalbin (red fluorescence) and (H) grp75 (green fluorescence). (I) The merged picture shows colocalization of calumenin and grp75 (yellow fluorescence). Each of the merged pictures were made with the same magnification as each of the individual stainings.

MRC-5 V2 cells do not express fibulin-1C in concentrations high enough for reliable detection of the antigen. Therefore, cells were transfected with fibulin-1C in order to reveal a putative co-localization with reticulocalbin and calumenin. As seen in Fig 7A reticulocalbin stains a perinuclear reticular network consistent with the ER localization [36] and fibulin-1C shows an overall similar localization (Fig 7B). When the pictures are merged the co-localization is apparent in the reticular network as seen by the yellow staining (Fig 7C). Thus, an interaction between fibulin-1C and reticulocalbin is possible in the ER. Similarly, calumenin being localized in the secretory pathway shows an abundant co-localization with fibulin-1C, which is apparent from the yellow staining when the pictures are merged (Fig 7F). Co-staining of MRC-5 V2 fibroblasts with anti-reticulocalbin and anti-grp75 revealed partial overlap as seen by the yellow fluorescence on the merged picture indicating that interaction between grp75 and reticulocalbin is possible in the ER (Fig 7I).

### Tissue localization of calumenin, reticulocalbin and fibulin

**Fig 8:**
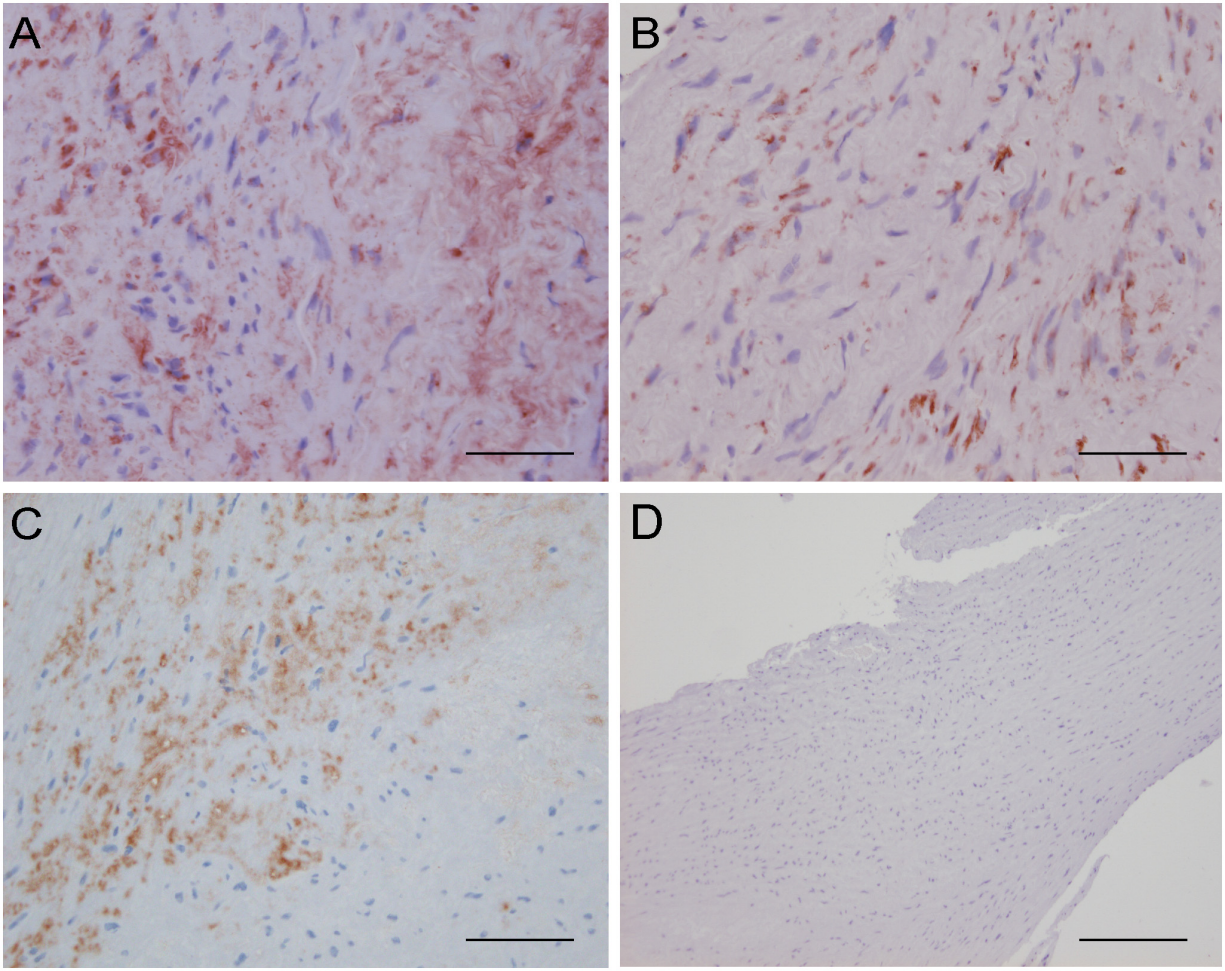
Immunohistochemistry of calumenin, reticulocalbin and fibulin in arterial tissue. (A) calumenin localization in arteria mammaria, (B) reticulocalbin localization in arteria mammaria, (C) fibulin-1 localization in arteria carotis and (D) negative control of arteria carotis. The scale bar is 50 μm in A, B and C and 200 μm in D.

Since fibulin binds strongly to calumenin and reticulocalbin we examined the localization in arterial tissue of all three proteins by immunohistochemistry (Fig 8). Calumenin shows a strong diffuse staining (Fig 8A) in agreement with intracellular localization as well as a substantial extracellular component as it is a secreted protein [14,15]. Reticulocalbin on the other hand shows a more discrete staining mainly localized near the nuclei (Fig 8B) probably due to its intracellular localization. The staining of fibulin-1 (Fig 8C) has a similar pattern to the calumenin staining. The latter observation would be in line with the two proteins being secreted as a complex because of their strong interaction although further studies are needed to firmly conclude this.

## Discussion

Calumenin and reticulocalbin belong to the family of multiple EF-hand proteins reported to be mainly localized in various parts of the secretory pathway [1]. Although the functional properties of the CREC proteins are steadily emerging there are still several unknown aspects. The primary function of the CREC proteins seems to be interaction with and regulation of a number of proteins in the ER and SR [2]. Thus, reticulocalbin and calumenin have been reported to interact with the Sec63p protein of the protein translocase [37]. Calumenin shows a Ca^2+^ dependent interaction with the ryanodine receptor-1 that takes part in the excitation contraction mechanism by releasing Ca^2+^ from the SR upon depolarization of the plasma membrane [26] and interacts with and inhibits the Ca^2+^-ATPase [38]. Some of the most well described functions seem to be that calumenin interacts with and inhibits the vitamin K dependent γ-carboxylation system that takes care of the synthesis of a number of the coagulation factors [39]. Recently, an extracellular function has been described that calumenin suppresses ERK1/2 signalling and cell migration by inhibiting proteolysis of fibulin-1 [32].

In order to further reveal novel functional aspects of the CREC family members one approach is to identify novel interacting proteins. We have previously identified serum amyloid P component [40] and thrombospondin-1 [33] as interacting partners with calumenin further linking this protein with the immunological defence system, haemostasis and/or thrombosis. Recently, we have shown that ERC-55 interacts with a wide variety of proteins in the secretory pathway as well as in the cytosol suggesting a broad spectrum of activities including immunity, redox homeostasis, cell cycle regulation and coagulation [5]. In the present report we have used a combination of affinity purification, immunoprecipitation and SRP analysis to identify two secretory proteins, fibulin-1C and C1 esterase inhibitor, as well as a cellular protein, grp75, that interact with both calumenin and reticulocalbin.

### Fibulin-1

From the placenta extract we purified by 1D- and 2D-PAGE a protein using steadily increasing concentrations of NaCl from around 0.2 M up to 1 M. The protein was identified as fibulin-1. Fibulin-1 is a member of the fibulin family of proteins consisting of eight members, fibulin-1 to fibulin-8 [41,42]. Fibulin-1 has four isoforms all containing an N-terminal signal sequence, three anaphylatoxin domains nine EGF-like modules as well as a C-terminal fibulin-type module. The four variants differ in their C-terminal. Fibulin-1A consists of the sequence that is mutual for all the variants. This sequence contains 537 amino acids and a signal sequence of 29 amino acids. It is the extra varying C-terminal sequence that determine the outcome of the three others variants. These three C-terminal sequences consist of 35, 117 and 137 amino acids for the B, C, and D variants, respectively [43]. The identified fibulin-1 is the C variant. Thus, in order to verify the interaction we expressed fibulin-1C as a GST fusion protein in E. coli for surface plasmon resonance verification. This confirmed that fibulin-1C interacts with reticulocalbin as well as with calumenin with a rather high affinity. The dissociation constant was estimated to be around 50 nM. Apparently, the interaction, at least with reticulocalbin, is not dependent upon the presence of Ca^2+^. Thus even though Ca^2+^ is bound to EF-hands in the CREC proteins and to EGF motifs in fibulin, any conformational changes induced by Ca^2+^ is not important for the strength of the interaction. Reticulocalbin may interact with fibulin-1C in the ER with a chaperone function while calumenin could serve a chaperone function in the ER but also interact through the whole secretory pathway and could also interact with fibulin in the extracellular space where the Ca^2+^ concentration is high.

Fibulin-1 is a secreted Ca^2+^-binding plasma and extracellular matrix protein that is induced by estrogens in ovarian cancer cells [44]. Fibulins possess diverse roles. They interact with several protein ligands, participates in elastic fiber biology and are differentially expressed in tumors [41]. Other studies implicate fibulin-1 in hemostatic and thrombotic events. Fibulin-1 has been found to interact with the plasma protein fibrinogen and to be incorporated into fibrin clots [45]. As an extracellular matrix protein present in vessel walls, fibulin-1 may act as a mediator of platelet adhesion via a bridge of fibrinogen during vascular injury. In this way, fibulin-1 is regarded as a thrombogenic component in the earliest events of thrombus formation [46]. Calumenin has been reported to be present in thrombotic plaques [30]. Our results have revealed that calumenin and fibulin-1 strongly interacts with dissociation constant in the nanomolar range. Calumenin and fibulin-1 may thus exist in complex through the whole secretory pathway as well as being secreted as a complex although direct observations are needed to firmly conclude this. Most recently, Wang et al. also found fibulin-1D to interact with calumenin and further that calumenin protects fibulin from degradation by matrix metalloproteinase. The result is an inhibition of cell migration through a suppression of the ERK1/2 signalling pathway [32].

### C1 esterase inhibitor

From the 2D-PAGE analysis we identified an acidic protein eluted at 0.2 M and 0.4 M NaCl. The protein was identified as C1 esterase inhibitor. C1 esterase inhibitor consists of 478 amino acids giving an expected molecular mass of approximately 53 kDa. From the gel separation it was estimated that the protein is about 80–100 kDa in size substantially higher than the expected mass. However, the protein is highly glycosylated (49%), which may explain the about twofold higher molecular mass when compared with the expected mass [47]. C1 esterase inhibitor is a serine protease inhibitor (serpin) that influences the activation of the complement system for the prevention of bacterial infections. In addition, C1 esterase inhibitor is the primary inhibitor of factor XIIa [48]. Deficiency of C1 esterase inhibitor causes hereditary angioedema (HAE), a disease characterized by returning oedema of the face, mouth or airway [48]. In order to verify the interaction we performed surface plasmon resonance studies with C1 esterase inhibitor. Thus, it interacts with reticulocalbin with a dissociation constant around 1 μM somewhat weaker than with calumenin where the dissociation constant is estimated to about 150 nM. The interaction, at least with calumenin is dependent upon Ca^2+^ in the mM range. With the concentrations of Ca^2+^ in the secretory pathway it seems possible that C1 esterase inhibitor may interact with reticulocalbin and calumenin mainly in the ER and with calumenin in the whole secretory pathway as well as extracellularly. C1 esterase inhibitor does not contain obvious Ca^2+^ binding domains, neither EF-hands nor EGF motifs, so it is likely that Ca^2+^-induced conformational changes in the CREC protein is responsible for the changes in the binding strength observed.

### Grp75

By immunoprecipitation we detected grp75 (or mortalin) as a putative interacting partner with reticulocalbin. Subsequent SRP analysis showed that grp75 interacted with calumenin as well as with reticulocalbin at rather high affinities with dissociation constants at the low nanomolar level.

Grp75 belongs to the chaperone heat shock protein (hsp70) family of proteins and in different studies it has been localized in the mitochondria, ER, plasma membrane, cytoplasmic vesicles and the cytosol [49]. Thus, with calumenin and reticulocalbin localized in the ER there seems to be a possibility for interactions here. Grp75 is differentially distributed in normal and transformed cells [50] and it has in several cases been observed in association with malignant transformation of cells.

### Conclusion

By a combination of affinity purification, immunoprecipitation and SRP analysis we identified three proteins, fibulin-1C, C1 esterase inhibitor and grp75 capable of interacting with two CREC proteins, calumenin and reticulocalbin. Fibulin-1C showed a strong reaction with both proteins with a *K*_D_ around 50 nM, and the binding was not particularly dependent upon the presence of Ca^2+^ at least in case of reticulocalbin. C1 esterase inhibitor interacts with reticulocalbin with an affinity around 1 μM and somewhat stronger with calumenin with an affinity at 150 nM. At least for calumenin, the binding was strongly dependent upon the presence of Ca^2+^ in the mM range. Grp75 was found to interact with the two CREC proteins with an affinity in the low nanomolar range.

These identified interactions largely confirm a number of previous discovered functions such as participation in chaperone activity, control of cell proliferation and cellular aging (grp75), modulation of cellular transformation and participation in haemostasis and thrombosis (fibulin-1), as well as modulation of the complement system in fighting bacterial infection (C1 esterase inhibitor).
