# Correction: Fibulin-1C, C1 Esterase Inhibitor and Glucose Regulated Protein 75 Interact with the CREC Proteins, Calumenin and Reticulocalbin

**DOI:** 10.1371/journal.pone.0139293

**Published:** 2015-09-23

**Authors:** Gry Aune Westergaard Hansen, Maja Ludvigsen, Christian Jacobsen, Claudia Cangemi, Lars Melholt Rasmussen, Henrik Vorum, Bent Honoré

The fifth author’s name is listed incorrectly within the citation. The correct name is Lars Melholt Rasmussen. The correct citation is: Aune Westergaard Hansen G, Ludvigsen M, Jacobsen C, Cangemi C, Rasmussen LM, Vorum H, et al. (2015) Fibulin-1C, C1 Esterase Inhibitor and Glucose Regulated Protein 75 Interact with the CREC Proteins, Calumenin and Reticulocalbin. PLoS ONE 10(7): e0132283. doi:10.1371/journal.pone.0132283
